# Membraneless Organelles and Organ Fibrosis: New Perspectives and Therapeutic Opportunities

**DOI:** 10.3390/cells15201872

**Published:** 2026-10-15

**Authors:** Xikai Liu, Yihao Zhou, Melanie Tran, Yuchen Chen, Dong Yang

**Affiliations:** 1 School of Biological and Chemical Engineering, Nanyang Institute of Technology, Nanyang 473004, China; xikai.liu@nyist.edu.cn; 2 College of Life Sciences, Wuhan University, Wuhan 430072, China; zzzyihao@outlook.com; 3 Division of Nephrology, Department of Medicine, University of Connecticut School of Medicine, Farmington, CT 06030, USA; metran@uchc.edu; 4 Division of Surgical Sciences, Department of Surgery, University of Virginia, Charlottesville, VA 22903, USA

**Keywords:** organ fibrosis, membraneless organelles, phase separation, biomolecular condensates, fibrotic pathway, therapeutic targeting

## Abstract

Organ fibrosis is a maladaptive repair outcome in which persistent injury drives extracellular matrix (ECM) accumulation, distortion of tissue architecture, and progressive loss of organ function. Although major fibrotic pathways are well established, it remains less clear how mechanical, inflammatory, oxidative, metabolic, and other stress signals are spatially organized and converted into persistent cell-state changes. Many biomolecular condensates, also known as membraneless organelles (MLOs), are organized through liquid–liquid phase separation (LLPS), which can alter signaling efficiency, transcriptional output, RNA handling, and stress responses. Increasing evidence links this mode of molecular organization to fibrosis-relevant pathways across the kidney, liver, cardiovascular system, and lung. In this review, we summarize current evidence for MLO-mediated regulation of organ fibrosis, discuss the potential to therapeutically modulate assembly, client recruitment, and material properties, and highlight key translational challenges, including in vivo causality, cell-type and disease-stage selectivity, delivery, and safety. Overall, MLOs provide a useful framework for understanding how fibrotic signals are organized and sustained, while also opening therapeutic opportunities beyond conventional single-node inhibition.

## 1. Introduction

Organ fibrosis represents a maladaptive wound-healing response in which persistent injury and failed resolution drive the progressive accumulation of extracellular matrix (ECM), distortion of tissue architecture, and eventual organ dysfunction [1,2]. Across organs, including the kidney, lung, liver, and heart, fibrotic remodeling emerges when chronic mechanical stress, sustained inflammatory activation, oxidative injury, and metabolic imbalance converge to maintain profibrotic cell states, such as activated myofibroblasts and maladaptive changes in parenchymal cells [3]. These processes are interconnected rather than independent. For example, metabolic reprogramming, including increased glycolytic activity, can support fibrogenic activation, alter redox and metabolite availability, and stabilize pathological cell states [3,4]. A key unresolved question is how these diverse and often transient inputs are organized within cells and converted into persistent signaling and transcriptional programs that sustain fibrosis.

**Figure 1:**
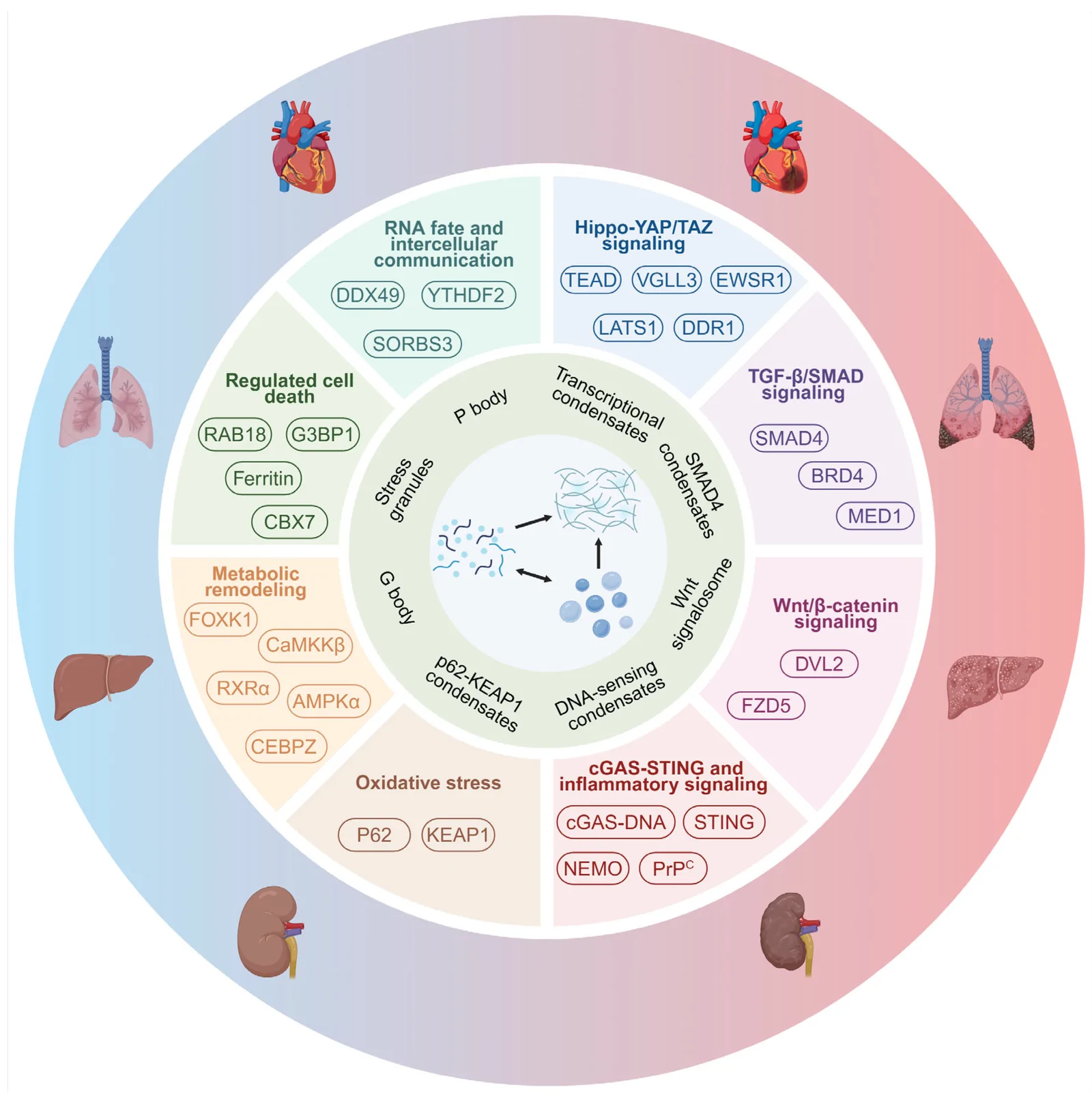
From MLOs to fibrosis-related signaling networks and organ fibrosis. This schematic summarizes how MLOs and condensate assemblies connect to organ fibrosis. The central circle depicts transitions from soluble biomolecules to liquid-like condensates and more stable condensate states. The inner ring lists representative MLOs and condensates, including stress granules, P bodies, G bodies, transcriptional condensates, SMAD4-associated condensates, Wnt signalosomes, DNA-sensing condensates, and p62-KEAP1 condensates. The outer ring shows fibrosis-relevant pathways and key molecules, including Hippo-YAP/TAZ signaling, TGF-β/Smad Family Protein (SMAD) signaling, Wnt/β-catenin signaling, cGAS-STING and inflammatory signaling, oxidative stress, metabolic remodeling, regulated cell death, RNA fate, and intercellular communication. In the outermost circle, representative organs are shown from healthier states on the left to fibrotic states on the right. The strength of direct evidence linking individual condensates to fibrosis in vivo varies among the examples shown. This figure provides a conceptual framework for understanding how phase separation may organize fibrogenic signaling across major organs. Created in BioRender. Yang, D. (2026) https://BioRender.com/yxyhfo4.

Biomolecular condensation provides one possible layer of this regulation. Membraneless organelles (MLOs) are cellular compartments that concentrate proteins and nucleic acids without a surrounding membrane [5,6,7]. Familiar nuclear examples include nucleoli, which support ribosome biogenesis, and Cajal bodies, which help assemble and mature RNA-processing complexes [8,9]. In the cytoplasm, P bodies participate in mRNA storage and turnover, whereas stress granules (SGs) assemble during stress and recruit untranslated mRNAs and RNA-binding proteins [10,11]. These established MLOs illustrate how cells organize biochemical activities without membranes; representative assemblies and their links to fibrosis are summarized in Figure 1.

Many MLOs were formed by the process of liquid–liquid phase separation (LLPS), in which proteins and nucleic acids self-assemble via weak but multivalent interactions to form dynamic compartments without a delimiting membrane [12,13]. Physiologically, phase separation occurs when a supersaturated liquid solution of biomacromolecules, primarily scaffold proteins and nucleic acids, spontaneously separates or de-mixes into two phases. This produces a low-concentration dilute phase and a high-concentration droplet-like dense phase that coexist stably [14]. Factors such as temperature, ionic strength, and pH act together to determine whether MLOs assemble or dissolve [15]. Importantly for fibrosis, oxidative stress, endoplasmic reticulum (ER) stress, DNA damage, osmotic changes, and mechanical strain can alter the assembly, composition, and material properties of MLOs and related condensates [16].

This mode of organization is relevant to fibrosis because many core signaling and transcriptional systems rely on multivalent protein-protein and protein-nucleic-acid interactions [10]. The Hippo-Yes-associated protein (YAP)/transcriptional coactivator with PDZ-binding motif (TAZ) condensates can organize mechanoresponsive transcription [17,18]; NF-κB essential modulator (NEMO) phase separation can concentrate components required for NF-κB activation [19]; and Sequestosome-1(p62)-kelch-like ECH-associated protein 1 (KEAP1)-nuclear factor erythroid 2-related factor 2 (NRF2) condensates can regulate oxidative-stress signaling through selective partitioning [20]. In chronically injured tissues, such compartmentalization may help convert fluctuating stress signals into sustained pathway activity and stable cell-state changes [17,21]. It also creates therapeutic possibilities beyond catalytic inhibition alone, because disease-relevant phase behavior may be altered by changing assembly thresholds, molecular interactions, client recruitment, or material state [22,23].

In this review, we first summarize the basic principles of LLPS, established MLOs, and fibrosis-related biomolecular condensates. We then organize current evidence around major fibrotic pathways and cellular processes, followed by a tissue-specific synthesis across major organs. Finally, we discuss therapeutic strategies for modulating MLOs and phase behavior, together with the experimental and translational challenges that must be addressed before these concepts can be applied to organ fibrosis.

## 2. Phase Separation and Organ Fibrosis-Related MLOs

Phase separation reflects a de-mixing transition when the sum of interactions between scaffold and client components—e.g., protein–protein, protein–RNA, and RNA–RNA—exceeds the percolation threshold [24]. The individual proteins and RNA will separate themselves from the surrounding environment, condensing into a concentrated phase that forms a membrane-free region. This concentrated phase, named biomolecular condensates, also known as MLOs [25], exhibits characteristics such as the ability to fuse with each other, having minimal surface tension, dynamic exchange with the surrounding corresponding soluble phase, and liquid-like properties [26]. These MLOs could undergo reconstitution in vitro under certain conditions and have important biological functions in vivo. In addition, MLOs are also associated with various diseases. This section briefly introduces these concepts before the fibrosis-specific mechanisms are discussed in Section 3.

### 2.1. LLPS In Vitro and in Cells

Before discussing phase separation in fibrosis, it is useful to clarify several related but non-equivalent terms. Biomolecular condensates and membraneless organelles (MLOs) are closely related and often overlapping concepts. Biomolecular condensates are membrane-free assemblies that concentrate selected biomolecules, whereas well-established MLOs, such as the nucleolus and stress granule, have characteristic compositions and biological functions and are usually referred to by their conventional organelle names. Under microscopy and reconstruction, condensates may appear as puncta, spots, or droplet-like structures; these are morphological descriptions in general [25]. MLOs and biomolecular condensates assemble through LLPS. Macromolecular clustering is a broader, mechanism-neutral term for the local enrichment of molecules and may arise through LLPS or other assembly processes. Protein aggregation generally refers to more stable and less dynamic assemblies. Under persistent stress or disease conditions, some liquid-like condensates may become less dynamic and transition toward gel-like or solid-like states, which in some contexts can contribute to protein aggregation or other stable macromolecular assemblies, including fiber-like assemblies [27].

LLPS occurs in cells continuously and can be reconstructed in vitro. Phase separation has been reconstructed in a test tube containing purified protein and RNA structures. The first reconstituted droplet by phase separation in vitro is the formation of actin polymers [28]. In cells, liquid-like MLOs formed by LLPS are generally regular round droplets that can be recovered after photobleaching [29]. Under excessive stress or recombinant protein overexpression, some condensates may become less dynamic and transition toward gel-like or solid-like states. Such material-state transitions are increasingly associated with disease-related aggregation and loss of normal compartment function [30,31].

### 2.2. Biological Functions of LLPS

By concentrating selected proteins, RNAs, and enzymes while excluding others, phase separation can accelerate biochemical reactions, organize signaling, regulate transcription, and buffer cellular stress [10,26]. Condensation also provides a rapid and reversible way to reorganize cellular components in response to changing conditions; at neuronal synapses, for example, phase-separated assemblies help organize signaling machinery [32]. In fibrosis, the same general principles are relevant to transcription, metabolic regulation, RNA handling, inflammatory signaling, and stress adaptation. Recent examples include retinoic acid receptor RXR-alpha (RXRα) condensates and ATP-dependent RNA helicase DDX49 (DDX49)-containing lipid-induced granules (LIGs) in liver fibrosis regulation [33,34], as well as m^6^A-dependent YTH domain-containing family protein 2 (YTHDF2) condensation in diabetic cardiac fibrosis [35].

Phase separation also contributes to genome organization and transcriptional control. Condensates can compartmentalize chromatin-associated factors, promote or restrict access to selected genomic regions, and concentrate transcriptional regulators at active loci [36,37,38]. These functions provide a new perspective on our understanding of cell fate determination and gene regulation during fibrotic disease development.

### 2.3. Organ Fibrosis-Related MLOs

The subcellular location of MLOs and condensates helps explain their functions [8,9,11,39,40,41,42,43]. Nuclear assemblies can regulate transcription and RNA processing, whereas cytoplasmic condensates can coordinate stress responses, metabolism, selective autophagy, RNA turnover, and cell-death pathways [41,44]. Membrane-associated condensates can organize receptor-proximal signaling and mechanotransduction [45]. This spatial organization is particularly relevant to fibrosis, where nuclear gene regulation, cytoplasmic stress adaptation, and extracellular-matrix sensing are tightly coupled.

Fibrosis-related MLOs and condensates have now been identified across these different cellular compartments. In the nucleus, Forkhead box protein K1 (FOXK1) forms condensates that recruit RNA polymerase II and promote transcription of glycolytic genes in tubular epithelial cells, contributing to metabolic reprogramming and renal fibrosis [46]. Similarly, baicalein suppresses nuclear CCAAT/enhancer-binding protein zeta (CEBPZ) phase separation, enhances tumor protein 53 (*TP53*) transcription, inhibits the p53/hexokinase 2 (HK2) glycolytic program, and promotes hepatic stellate cell senescence, thereby attenuating liver fibrosis [47].

Cytoplasmic condensates can regulate inflammatory signaling, RNA fate, and stress responses. In proximal tubular epithelial cells, cellular prion protein (PrPC) undergoes phase separation and activates TANK-binding kinase 1 (TBK1)-interferon regulatory factor 3 (IRF3) signaling, promoting profibrotic responses in tubular cells and fibroblasts; PrPC loss or TBK1 inhibition attenuates renal fibrosis [48]. In steatotic hepatocytes, DDX49 forms LIGs that sequester m^5^C-modified tissue inhibitor of metalloproteinase 2 (*TIMP2*) mRNA and reduce production of the profibrotic hepatokine TIMP2, thereby limiting fibrosis progression [34].

At the plasma membrane, phase separation can organize mechanosensitive signaling. Matrix stiffness or collagen binding promotes epithelial discoidin domain receptor 1 (DDR1) phase separation and co-condensation with large tumor suppressor homolog 1 (LATS1) in vascular smooth muscle cells. DDR1/LATS1 condensates disrupt the LATS1-MOB1 interaction, reduce Hippo pathway activity, activate YAP, and promote arterial ECM remodeling and stiffening [49]. These examples illustrate how the location of a condensate can shape its biological output, from transcriptional control in the nucleus to signaling and RNA regulation in the cytoplasm and mechanotransduction at the plasma membrane. The representative fibrosis-associated MLOs and biomolecular condensates discussed in this review are summarized in Table 1. To complement the pathway-based organization of Section 3, the entries are arranged by subcellular localization.

## 3. Mechanistic Landscape of Phase Separation in Organ Fibrosis

Fibrosis develops through the combined effects of mechanical stress, inflammatory signaling, oxidative injury, metabolic changes, and repeated cell damage. Phase separation is relevant to this process because it can bring signaling proteins, transcription factors, RNAs, and enzymes into the same local compartment and thereby change how strongly or how long a pathway is active. In this sense, biomolecular condensates are better viewed as a way of organizing fibrotic signaling than as a separate profibrotic pathway. Here, we summarize MLOs and condensates connecting to fibrosis-relevant signaling pathways in Figure 1.

Importantly, condensates are not always harmful. Depending on their components, cellular location, and duration, they may strengthen a disease-promoting signal, limit an excessive response, or help cells adapt to stress. To connect biophysical condensate behavior to these canonical networks, the sections below follow the major signaling pathways and cellular processes that are commonly involved in fibrosis, while distinguishing direct evidence derived from fibrotic tissues from broader mechanistic studies on phase separation.

### 3.1. Hippo-YAP/TAZ Signaling and Mechano-Transduction Axis

Mechanical stress is a central feature of fibrosis. As ECM accumulates, tissue matrix stiffness, stretch, and cytoskeletal tension increase, and these changes can further activate fibroblasts and other fibrogenic cells. The YAP/TAZ signaling pathway is a major link between these mechanical cues and gene expression, making it one of the clearest places where condensate biology may intersect with fibrosis.

Multiple studies have firmly established that condensates encode and drive YAP/TAZ transcriptional activity. Under hyperosmotic stress, YAP rapidly forms dynamic nuclear condensates that reorganize genome topology and concentrate transcription-associated factors [17]. TAZ can also form nuclear condensates that recruit TEA domain family member 4 (TEAD4), bromodomain-containing protein 4 (BRD4)/mediator of RNA polymerase II transcription subunit 1 (MED1), and cyclin-dependent kinase 9 (CDK9) and support transcriptional activation. The CC domain is crucial for the phase separation function of TAZ. Deletion of the TAZ CC domain prevents the formation of TAZ condensates and its ability to induce the expression of TAZ-specific target genes [18]. More recent work suggests that TEAD proteins themselves can form transcriptional condensates and recruit YAP/TAZ, helping define that TEAD condensates serve as central organizing hubs to dynamically concentrate active YAP and other markers of transcriptional activation within the nucleus [64]. At the upstream end of the pathway, collagen and matrix stiffness induce DDR1 phase separation and co-condensation with LATS1, which reduces Hippo pathway restraint and promotes YAP activation [49,65]. Together, these studies provide a direct conceptual link from the ECM to condensate assembly and downstream transcription. Fibrosis-focused studies further support a link between mechanosensitive condensate formation and profibrotic transcription. A recent pulmonary fibrosis study reports that circELP2 promotes tripartite motif-containing protein 25 (TRIM25) phase separation, which enhances ubiquitin-dependent degradation of 14-3-3ζ and promotes YAP1 nuclear translocation. Nuclear YAP1 subsequently activates cytoskeleton-remodeling genes and increases fibroblast-to-myofibroblast differentiation [59]. A similar link between mechanical cues and condensate formation has also been reported in cardiac fibrosis. Vestigial-like protein 3 (VGLL3) is a TEAD-binding transcription cofactor and has been identified as a YAP-regulated gene in the breast cancer context, although a direct YAP/TAZ-VGLL3 link has not been established in the heart [66]. In cardiac myofibroblasts, matrix stiffness promotes nuclear entry of VGLL3 and its incorporation into non-POU domain-containing octamer-binding protein (NONO)/ewing sarcoma breakpoint region 1 protein (EWSR1)-containing condensates. VGLL3 interacts with EWSR1 in these condensates and suppresses miR-29b, which normally targets collagen mRNA. Consistent with this mechanism, *Vgll3* deficiency reduces fibrosis after myocardial infarction [50]. These findings make mechanotransduction one of the strongest examples of phase separation being connected to an organ-level fibrotic phenotype.

### 3.2. TGF-β/SMAD Signaling and Transcriptional Control

TGF-β/SMAD signaling is one of the best-established drivers of fibrosis across organs. It promotes myofibroblast activation, ECM production, cell-cycle changes, senescence, and other long-lasting changes in cell state. Because much of SMAD signaling is carried out through transcriptional complexes in the nucleus, condensate formation offers a possible way to control how much SMAD is available for transcription and how efficiently transcriptional machinery is recruited.

Current studies suggest at least two ways in which phase separation can influence this pathway. First, condensates can reduce signaling by sequestering an essential pathway component. SMAD4 can be retained within splicing factor proline/glutamine-rich (SFPQ) condensates, thereby limiting the cellular response to TGF-β; disrupting the phase-separation capacity of SFPQ increases TGF-β sensitivity [67]. Second, transcriptional condensates can support sustained gene expression by concentrating coactivators and transcriptional machinery. BRD4 and MED1 can form condensates at super-enhancers, providing a general mechanism by which transcriptional regulators are concentrated at active chromatin sites. BRD4 and MED1 rely on their intrinsically disordered regions (IDRs) to form droplets, and in vitro reconstruction experiments and truncated mutant experiments have shown that MED1-IDR droplets can compartmentalize and concentrate the transcription apparatus from nuclear extracts [38]. A recent study provides a more organ-fibrosis-focused example: TGF-β1 enhances heterogeneous nuclear ribonucleoprotein L (hnRNP L) phase separation in lung myofibroblasts. TGF-β1 promotes hnRNP L phase separation in lung myofibroblasts. Mutational analysis identified residues 26–111 and 274–374 as important for condensate formation, and disrupting this behavior reduced profibrotic transcription and fibroblast-to-myofibroblast differentiation in vitro and in vivo [51]. In fibrosis, this type of organization could help maintain profibrotic transcription after SMAD complexes reach permissive chromatin. Direct evidence that specific SMAD-containing condensates are required for fibrosis in vivo is still limited, so this remains an important area for future work.

### 3.3. Wnt/β-Catenin Signaling

Wnt/β-catenin signaling is repeatedly implicated in fibroblast activation, epithelial injury responses, and maladaptive repair across organ systems. Unlike pathways that are organized mainly in the nucleus, Wnt signaling begins with membrane-associated signalosomes, and their assembly can also be controlled by phase separation.

Disheveled-2 (DVL2) undergoes phase separation in the cytoplasm and is recruited by the signalosome—whose components are Frizzled-5 (FZD5) and Axis inhibition protein 1 (Axin-1)—at the plasma membrane after Wnt stimulation, promoting Wnt receptor signalosome formation and disassembly of the cytosolic β-catenin destruction complex [68]. Post-translational modification (PTM) provides an additional level of control: WW domain-containing protein 2 (WWP2)-mediated K63-linked ubiquitylation increases DVL2 multivalency and promotes condensate formation, which strengthens Wnt signaling and β-catenin stabilization [69]. These studies show how phase separation can regulate the efficiency of Wnt signaling. However, compared with the YAP/TAZ field, direct evidence that DVL2 condensation is required for fibrosis in vivo remains limited.

### 3.4. cGAS-STING and DNA-Sensing Responses

DNA damage, mitochondrial dysfunction, and cellular senescence can release DNA into the cytosol and activate the cGAS-STING pathway. This pathway is increasingly linked to chronic inflammation and fibrosis, and both the sensor and downstream signaling complex show condensate-like behavior.

DNA can trigger cGAS phase separation, and the resulting cGAS-DNA droplets increase local interactions that support cGAMP production. The positively charged, disordered N-terminus of cGAS strengthens these multivalent interactions, while longer DNA and free zinc further promote condensation and enzyme activity [70]. cGAS-DNA condensates can also limit access of the exonuclease three-prime repair exonuclease 1 (TREX1), thereby changing how quickly cytosolic DNA is degraded [71]. Downstream, STING can form ER-associated condensates after DNA-virus infection or cGAMP treatment. These structures sequester STING, TBK1, and cGAMP and appear to limit excessive pathway activation, acting as a buffering step rather than a simple amplifier [72]. The cGAS-STING system therefore illustrates an important general point: condensates can strengthen signaling at one step and restrain it at another. Importantly, the pathway itself has direct relevance to renal fibrosis: global *cGAS* deficiency and global or myeloid cell-specific *STING* deletion reduce kidney inflammation and fibrosis, with pharmacologic inhibition producing a similar protective effect [73]. These findings establish a causal role for cGAS-STING signaling in renal fibrosis, although they do not yet show that the fibrotic phenotype depends on cGAS/STING phase separation. Direct phase separation-specific evidence in fibrotic organs remains less developed than the broader evidence linking cGAS-STING signaling itself to fibrosis.

### 3.5. Inflammatory Signaling Condensates

Chronic inflammation is a major driver of fibrosis and connects tissue injury to fibroblast activation. Many inflammatory pathways are built from multivalent interactions among ubiquitin chains, adaptor proteins, and kinases, which makes them well suited to condensate-based organization. In the NF-κB pathway, K63-linked and linear polyubiquitin chains can drive phase separation of the NF-κB essential modulator (NEMO). The resulting condensates concentrate signaling components needed for IKK activation, and disease-associated NEMO mutations that impair condensation also reduce signaling [19]. M1-linked ubiquitin chains similarly promote NEMO assembly after IL-1β stimulation, showing that ubiquitin-chain structure can help set the threshold for condensate formation and pathway activation [74].

A more direct fibrosis example comes from the kidney. PrPC is increased in proximal tubular epithelial cells during chronic kidney disease and can undergo phase separation. PrPC condensation activates TBK1-IRF3 signaling and promotes profibrotic responses in tubular cells and fibroblasts. Amlexanox, an FDA-approved TBK1 inhibitor, limited renal fibrosis and functional decline in mice both when given before fibrosis developed and when given after it was already established [48].

### 3.6. Oxidative Stress and NRF2 Signaling

Oxidative stress and mitochondrial dysfunction are common in fibrotic tissues. Cells respond to this stress through antioxidant pathways, selective autophagy, and SG assembly, and all three can involve condensate formation. The p62-KEAP1-NRF2 system provides a well-studied example. Notably, p62-dependent droplets can recruit KEAP1, NRF2, and polyubiquitin and thereby influence the balance between proteasomal degradation, autophagy, and NRF2 activation [20]. The neighbor of BRCA1 gene 1 protein (NBR1) further promotes p62 droplet formation and supports p62-dependent NRF2 activation by increasing phosphorylated p62 within these bodies [75]. Importantly, prolonged or poorly cleared condensates may become harmful. When autophagy is impaired, excessive retention of KEAP1 in p62 bodies can produce persistent NRF2 activation and liver injury, whereas p62 mutants with reduced KEAP1 binding lessen this effect [76]. Thus, condensates can support an adaptive antioxidant response but may also contribute to persistent stress signaling when their turnover is disturbed.

The cytoskeleton can also undergo oxidative stress-dependent phase separation. Oxidative stress converts vimentin filaments into small, reversible condensate-like particles through a process that depends on the single vimentin cysteine residue C328, and these particles can return to filaments after stress is removed [56]. Liquid-like vimentin condensates can also wet actin stress fibers and protect them from depolymerization, linking phase behavior to force-bearing cytoskeletal structures during fibrogenic remodeling [77].

### 3.7. Metabolic Remodeling

Metabolic reprogramming is an important part of fibrosis. In the kidney, FOXK1 is increased in fibrotic tissue and forms nuclear condensates by interacting with RNA polymerase II to drive the transcription of target genes in tubular epithelial cells. Disrupting FOXK1 condensates with 1,6-hexanediol or phase-separation-deficient IDR mutants reduced glycolytic gene expression. The in vivo suppression of FOXK1 condensate function attenuated renal fibrosis [46]. This study provides a direct example of how a transcriptional condensate can connect metabolic reprogramming to fibrosis.

The liver provides several additional examples. RXRα forms condensates with calcium/calmodulin-dependent protein kinase beta (CaMKKβ) and 5′-AMP-activated protein kinase (AMPKα) after treatment with K-80003, promoting AMPK activation, mitophagy, and reduced HSC activation [33]. Baicalein suppresses CEBPZ phase separation, increases TP53 transcription, reduces the p53/HK2 glycolytic program, and promotes HSC senescence [47]. With-no-lysine kinase 1 (WNK1) interacts with triosephosphate isomerase 1 (TPI1) within condensates, thereby enhancing glycolytic signaling and HSC activation [62]. Together, these examples show that condensate-dependent metabolic control can influence both mitochondrial quality and fibrogenic cell state.

Metabolism can also act upstream of condensate formation. In senescent cells, methyltransferase-like protein 3 (METTL3)-dependent chromatin changes increase HK2 expression, and the resulting metabolic reprogramming promotes stress-granule formation and reduces translation of selected cell-cycle mRNAs [78]. This work was not performed as an organ-fibrosis study, but it provides a useful mechanistic example of how metabolic state and condensate assembly can reinforce each other.

### 3.8. Regulated Cell Death

Cellular stress ultimately influences whether injured cells survive, adapt, or undergo regulated cell death. This is particularly important in fibrosis because the effect of cell death depends strongly on cell identity. Elimination of activated matrix-producing cells can favor fibrosis resolution, whereas death of parenchymal cells can prolong tissue injury and promote further fibroblast activation.

In activated HSCs, diallyl trisulfide (DAT) induces RAB18 phase separation, inhibits lipophagy, and promotes cuproptosis, leading to reduced liver fibrosis [60]. A separate study also places LLPS upstream of liver fibrosis in an antifibrotic HSC response [47]. Here, cell death is antifibrotic because it removes activated matrix-producing cells.

By contrast, when condensate-regulated cell death affects parenchymal or vascular cells required for tissue integrity and repair, it can increase injury and promote maladaptive remodeling. During ventilation-induced pulmonary fibrosis, nuclear receptor coactivator 4 (NCOA4) promotes ferritin phase separation, ferritinophagy, and ferroptosis in alveolar epithelial cells. Ferritin-containing extracellular vesicles released from injured epithelial cells are then taken up by fibroblasts and promote ECM accumulation [57]. A similar principle has been reported in the aged heart after myocardial ischemia–reperfusion injury. Chromobox protein homolog 7 (CBX7) forms condensates with the copper transporter ATPase 1 (ATP7A), retaining ATP7A intracellularly and reducing copper efflux, which promotes cuproptosis. Cuproptosis-related cell loss is concentrated in cardiomyocytes and capillary endothelial cells, whereas disruption of CBX7-ATP7A condensation restores ATP7A trafficking, suppresses cuproptosis, and reduces adverse remodeling and fibrosis [54].

Stress granules (SGs) also provide a clear example of this context dependence. SGs are ribonucleoprotein (RNP) granules that form during cellular stress and temporarily reduce translation. In an atrial fibrillation model, ras GTPase-activating protein-binding protein 1 (G3BP1)-associated SG formation reduced oxidative stress, Ca^2+^ overload, cardiac fibroblast proliferation, and collagen production, suggesting a protective stress-buffering role [53]. In contrast, combined polyhexamethylene guanidine phosphate (PHMG-p) exposure and respiratory syncytial virus infection in human lung organoids increased eukaryotic translation initiation factor 2 subunit 1 (eIF2α) phosphorylation and SG formation together with DNA damage and fibrotic gene expression [79]. These observations suggest that the effect of SGs may depend on the type, strength, and duration of stress rather than on SG formation alone.

### 3.9. RNA Fate and Intercellular Communication

Condensates can also regulate fibrosis through RNA fate. In steatotic hepatocytes, DDX49-containing lipid-induced granules (LIGs) sequester m^5^C-modified *Timp2* mRNA and reduce production of the profibrotic hepatokine TIMP2 [34]. In diabetic cardiac fibrosis, m^6^A-dependent YTHDF2 condensation promotes NOTCH1 downregulation and mitochondrial fission [35]. These studies illustrate how RNA partitioning or turnover can alter both extracellular signaling and intracellular cell-state programs.

Phase behavior may also affect communication between tissues. After overtraining, muscle-derived small extracellular vesicles enriched in lactylated sorbin and SH3 domain-containing protein 3 (SORBS3) have been reported to promote hepatocyte apoptosis through the MCL1-BAX/BAK axis and worsen liver fibrosis. Lactylation promotes SORBS3 phase separation into so-called ‘lactate bodies’, suggesting that condensate formation may influence the composition or activity of vesicle cargo [61]. This is an interesting extension of condensate biology from intracellular organization to cell-to-cell and inter-organ signaling.

### 3.10. Cross-Organ Comparison of Condensate-Mediated Fibrosis and Evidence Gaps

Across the studies reviewed, condensate-related mechanisms have been investigated in different organs and cellular settings. In the kidney, studies of FOXK1 and PrPC link tubular glycolytic reprogramming and inflammatory signaling, respectively, to renal fibrosis [46,48]. In the liver, condensate biology has been studied in HSC activation and survival, where it can either promote or limit fibrosis [32,34,60,62], as well as in hepatocyte-derived signaling, endothelial inflammation, and muscle–liver communication [34,52,61]. Cardiac and vascular studies connect VGLL3 and DDR1 condensation to matrix-dependent collagen production and arterial stiffening, respectively [49,50], while other work has examined RNA regulation in diabetic cardiac fibrosis and copper-dependent cell injury after ischemia–reperfusion in aged hearts [35,54]. In the lung, phase-separation-related mechanisms have been linked to epithelial polarity, ventilation-induced epithelial injury and fibroblast activation, and fibroblast-to-myofibroblast differentiation [51,57,58,59].

Taken together, these studies show that condensate biology intersects with fibrosis at multiple levels, but the depth of evidence remains uneven across organs and disease models. Importantly, findings in one organ cannot necessarily be assumed to apply through the same phase-separation-dependent mechanism in another. For example, VGLL3 knockdown reduces collagen expression in both cardiac myofibroblasts and HSCs isolated from fibrotic mouse livers, but the liver experiments did not establish whether this effect depends on phase separation [50]. Conversely, YAP nuclear condensates have been observed in mouse renal medullary cells, but their role in renal fibrosis has not been directly tested [17]. These examples highlight an important distinction: a protein may contribute to fibrosis in multiple organs without using the same condensate-dependent mechanism, and the presence of a condensate in a tissue does not by itself establish a role in fibrosis. Future cross-organ studies should therefore directly test whether phase separation is functionally required in the relevant cell types and injury models before extending a mechanism from one fibrotic disease to another.

## 4. Therapeutic Implications: Targeting Phase Separation

The therapeutic value of phase-separated biology lies in the possibility that disease-relevant molecular organization may be reversible. Rather than treating all condensation as pathological, therapeutic strategies should aim to selectively alter MLOs or phase-separated assemblies that sustain harmful signaling while preserving adaptive compartments such as stress-responsive MLOs. This can potentially be achieved by changing protein abundance, disrupting or stabilizing multivalent interactions, modifying post-translational regulation, altering client recruitment, or changing condensate material properties. However, most compounds reported to modulate phase separation were developed in cancer, infection, or neurodegenerative disease models, and direct antifibrotic evidence remains limited. Several fibrosis-focused studies already illustrate complementary intervention strategies. K-80003 promotes RXRα-CaMKKβ-AMPKα condensation and mitophagy in HSCs [33]; baicalein suppresses CEBPZ phase separation and shifts the TP53/HK2 program toward HSC senescence [47]; diallyl trisulfide (DAT) induces RAB18 phase separation and cuproptosis in activated HSCs [60]; and δ-Amyrenone (δAe) disrupts pathogenic CBX7-ATP7A condensation in aged ischemic hearts [54]. By contrast, amlexanox inhibits the downstream TBK1-IRF3 pathway activated by PrPC condensation rather than directly targeting condensate formation [48]. Table 2 summarizes these fibrosis-relevant examples together with a limited number of broader studies that illustrate general therapeutic principles.

### 4.1. Small Molecule Compounds

Small molecules provide one of the most practical approaches for modulating disease-associated phase behavior because they can alter protein conformation, molecular interactions, post-translational regulation, or downstream signaling. In fibrosis, depending on the biological role of a condensate, a small molecule may promote a protective assembly, disrupt a pathogenic one, alter its composition or material properties, or inhibit a downstream pathway that depends on condensate formation.

Several recent studies provide evidence consistent with a causal contribution in fibrotic disease models. In liver fibrosis, baicalein, a small molecule that directly binds CEBPZ, suppresses CEBPZ phase separation, increases TP53 transcription, reduces HK2-dependent glycolysis, and promotes HSC senescence [47]. iBET151, a pharmacological BRD4 inhibitor, suppresses PML/BRD4 condensate-associated super-enhancer activity and reduces hepatic inflammation and fibrosis [52]. Similarly, WNK-IN-11, a selective WNK1 inhibitor, inhibits WNK1 activity and suppresses WNK1-TPI1 condensate-associated glycolytic signaling in HSCs, thereby reducing the following profibrotic output [62]. Together, these studies show that suppressing pathogenic condensate activity can attenuate fibrosis by disrupting both transcriptional and metabolic programs that sustain fibrogenic responses. By contrast, therapeutic benefit can also be achieved by promoting specific condensate formation. K-80003 promotes formation of RXRα-CaMKKβ-AMPKα condensates, enhancing AMPKα activation and mitophagy and suppressing HSC activation and liver fibrosis [33]. DATS provides another example in which promoting phase separation is beneficial: DATS enhances RAB18 condensation, inhibits lipophagy, and promotes cuproptosis in activated HSCs, resulting in antifibrotic effects [60].

In the aged heart, δ-Amyrenone disrupts CBX7-ATP7A condensation, restores ATP7A trafficking and copper efflux, and improves cardiac repair while reducing fibrosis and arrhythmias [54]. In renal fibrosis, amlexanox attenuates fibrosis by inhibiting TBK1 downstream of PrPC phase separation [48]. Importantly, amlexanox acts as a downstream pathway inhibitor rather than a direct modulator of PrPC condensation, illustrating that small-molecule strategies can target either the condensate itself or a signaling dependency created by condensate formation.

**Figure 2:**
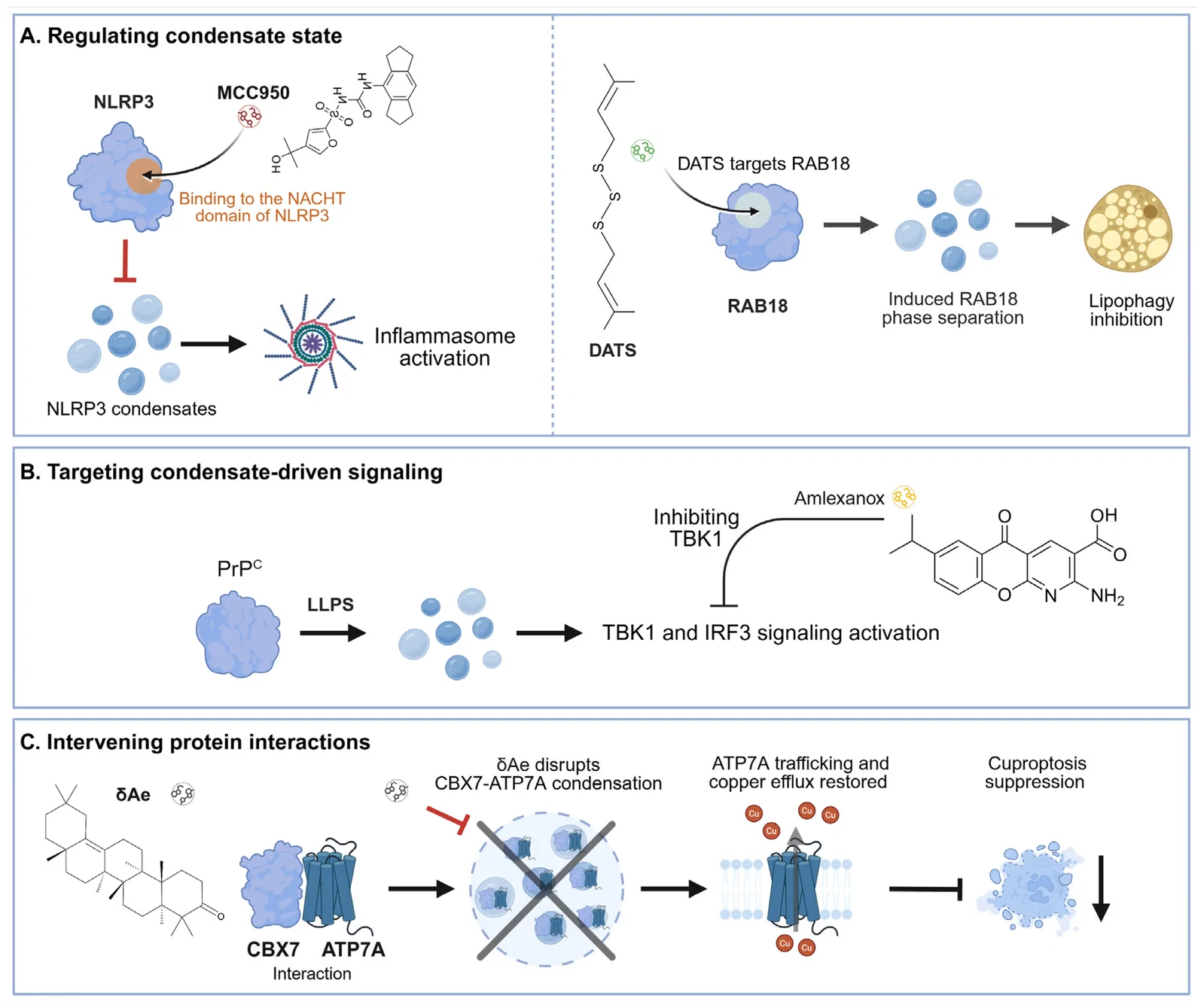
Pharmacological strategies for targeting condensate-related mechanisms in fibrosis-associated diseases. The figure includes three representative therapeutic strategies related to MLOs and phase separation. (**A**) Direct modulation of condensate formation. MCC950 directly binds the NACHT domain of NLRP3 and prevents NLRP3 condensation, thereby blocking inflammasome activation. DATS acts on RAB18 and promotes RAB18 phase separation, inhibiting lipophagy in liver fibrosis. (**B**) Targeting condensate-dependent downstream signaling. PrPC phase separation activates the TBK1-IRF3 inflammatory signaling axis. Amlexanox inhibits TBK1 and suppresses this pathway. (**C**) Intervening protein interactions. In aged myocardial IR injury, CBX7 forms LLPS condensates with ATP7A, causing ATP7A retention, impairing copper efflux, and promoting cuproptosis. δ-Amyrenone disrupts CBX7-ATP7A condensation, restores ATP7A trafficking and copper efflux, and improves cardiac repair with reduced fibrosis. These examples highlight complementary strategies for pharmacologically modulating condensate-related mechanisms for antifibrotic therapy. Created in BioRender. Yang, D. (2026) https://BioRender.com/chbavrt.

Beyond fibrosis, small-molecule studies in other disease models provide additional proof-of-principle that condensate assembly and material properties are pharmacologically tractable. For example, MCC950 suppresses NLRP3 phase separation and inflammasome activation [80], whereas other compounds can alter stress-granule assembly or dissolution [81,82]. G3BP inhibitor molecules (G3Ia and G3Ib) bind G3BP1/2 and can inhibit stress-granule formation or dissolve pre-existing SGs across several stress conditions [82]. These examples are not direct antifibrotic evidence but support the broader feasibility of pharmacologically controlling condensate behavior. Based on regulatory strategies, we summarize three representative therapeutic strategies for targeting condensates in fibrosis-associated diseases in Figure 2.

### 4.2. Peptide-Based Approaches

Peptides can provide greater sequence and interaction specificity than broadly acting small molecules and may therefore be useful for disrupting or stabilizing selected condensate interfaces. Rather than changing total protein concentration, peptide-based approaches can be designed to block interaction motifs, compete for binding surfaces, or interfere with recruitment of key clients. This strategy is attractive for phase-separating proteins because many condensates depend on repeated low-affinity interactions that may be selectively perturbed.

Current examples remain largely outside fibrosis. A cell-penetrating peptide that disrupts cellular nucleic acid-binding protein (CNBP)-SWI/SNF-related matrix-associated actin-dependent regulator of chromatin subfamily C member 2 (SMARCC2) interactions and CNBP condensation suppresses neuroblastoma growth [83], and inhibitory peptides targeting lactylated SPTAN1 have shown activity in HBV-related hepatocellular carcinoma [84]. These studies provide useful design principles, but analogous peptide strategies have not yet been established for the known major fibrosis-associated condensates.

Future therapeutic development will first require stronger causal evidence that a specific condensate contributes directly to fibrosis. Phase-separation-deficient mutants, rescue experiments comparing wild-type and LLPS-deficient variants, and chemically inducible condensate systems can help determine whether LLPS acts as an upstream driver, a signal amplifier, or a secondary consequence of fibrotic pathway activation. Progress will then depend on pairing this mechanistic evidence with selective interventions that alter condensate assembly, composition, or material properties without broadly disrupting physiological MLOs. Although condensate-directed therapy remains an emerging concept in fibrosis, advances in structure-guided peptide design, high-throughput screening, targeted delivery systems, and AI-assisted target discovery may help move the field toward fibrosis-specific therapies.

## 5. Discussion, Future Perspective, and Challenges

Research on MLOs and phase separation has expanded rapidly, but their role in organ fibrosis remains an emerging area. Although a growing number of studies link phase-separated assemblies to canonical fibrotic pathways, much of the mechanistic evidence still comes from purified systems or cultured cells, and only a limited number of studies directly demonstrate that altering phase behavior changes fibrosis in vivo. Establishing causality will require approaches that can manipulate phase separation independently of changes in protein abundance or conventional protein function. Optogenetic and chemically inducible systems, such as OptoDroplet and ChemDrop, combined with single-cell multi-omics and proteomic approaches, may help determine whether LLPS acts as an upstream driver of persistent profibrotic transcriptional programs. Complementary experiments should selectively disrupt domains required for phase separation and include rescue with wild-type versus LLPS-deficient variants. Pharmacological inhibitors or activators may provide additional support when their molecular targets and effects on condensate behavior are clearly defined. The key challenge is therefore not simply to identify additional condensates, but to establish which assemblies are mechanistically required for disease, in which cell types they act, and at what stage of fibrosis their activity becomes functionally important. Addressing these questions will require integration of mechanistic perturbation with primary-cell, organoid, and in vivo models, rather than relying on condensate formation alone as evidence of disease relevance.

Rigorous evidence is particularly important because not every punctum observed by microscopy represents a phase-separated condensate. Strong evidence usually combines several features, such as concentration dependence, fusion, rapid molecular exchange or fluorescence recovery, in vitro reconstitution, and mutants that selectively disrupt phase behavior without simply removing the protein [14]. This point is particularly important in fibrosis, where many candidate proteins already have well-known functions independent of phase separation. Ideally, future studies should show that a phase-separation-deficient mutant changes fibrosis while preserving other major functions of the protein.

Another unresolved question is whether chronic fibrotic stress changes the material properties of disease-relevant condensates over time. Studies outside the fibrosis field provide important precedents. Under oxidative stress, superoxide dismutase 1 (SOD1) can undergo LLPS and subsequently mature toward solid-like oligomers and aggregates, a process accelerated by disease-associated mutations [86]. Similarly, α-synuclein forms liquid condensates that can progress toward amyloid states, while β-synuclein can enter these condensates and slow their liquid-to-solid transition [27]. These findings raise the possibility that prolonged oxidative, metabolic, or inflammatory stress in fibrotic tissues may alter not only condensate formation but also condensate dynamics and reversibility. Whether similar liquid-to-gel or liquid-to-solid transitions occur during chronic organ fibrosis, and whether they contribute to persistent fibrotic signaling, remains to be determined.

Cell type and disease stage are likely to be equally important. The same phase-separation event may have different consequences in fibroblasts, parenchymal cells, endothelial cells, or immune cells, and a condensate that is adaptive during acute injury may become maladaptive when sustained chronically. This context dependence is already evident from fibrosis studies in which condensate formation can either promote disease, such as through profibrotic transcription or parenchymal-cell injury, or limit disease by enhancing stress adaptation, mitochondrial quality control, or selective elimination of activated matrix-producing cells. Accordingly, future studies should define condensate composition and dynamics in a spatially and temporally resolved manner. Single-cell and spatial transcriptomic approaches, quantitative imaging, and spatial metabolomic or proteomic technologies may help determine where and when disease-relevant condensates emerge and how their molecular composition changes during fibrosis progression [87,88].

Classifying condensates as beneficial or harmful is conceptually appealing, but this distinction is often context-dependent and difficult to make. A general precedent comes from cancer, where some tumor cells show an enhanced capacity to form stress granules; this stress-buffering response may support tumor-cell survival while worsening disease and harming the host [89]. A similar context-dependent principle also applies to organ fibrosis. During early injury and inflammation, liquid-like condensates may exert protective or adaptive functions by buffering stress, organizing transient repair signaling, or maintaining cellular homeostasis [16]. However, as fibrosis progresses toward fibroblast activation, extracellular matrix accumulation, and disease progression, persistent pathological stress may gradually disrupt condensate regulation. Under these circumstances, condensates may undergo liquid-to-gel or liquid-to-solid transitions, recruit profibrotic clients, and amplify fibrotic signaling, thereby contributing to fibrosis progression. Direct in vivo evidence defining the stage-specific roles of condensates in organ fibrosis remains limited. Future studies will therefore require standardized approaches for characterizing condensate formation, dynamics, composition, and material state. Resolving this distinction is also essential for deciding which condensates should be targeted, in which cell types, and at what stage of disease.

These biological uncertainties translate into therapeutic challenges. There is a lack of drugs for organ fibrosis and still tremendous challenges in developing effective phase-separation-modifying treatments for organ fibrosis. Several barriers need to be addressed before condensate-modulating strategies can be translated clinically, including target specificity, tissue delivery, pharmacokinetics, and off-target toxicity. Many small-molecule modulators have limited tissue selectivity or are rapidly cleared, and interference with proteins that participate in physiological MLOs may disrupt normal cellular functions in non-target organs [90,91,92]. Improving organ-, cell-, and subcellular-level delivery may therefore be particularly important. For example, functionalized triphenylphosphonium (TPP^+^)-containing nanoparticles have been used to deliver DRP1 inhibitors selectively to tubular mitochondria, reducing renal fibrotic injury while limiting off-target neurotoxicity [93].

Because fibrosis is driven by interconnected mechanical, metabolic, inflammatory, and stress-response pathways, modulation of a single condensate may not always be sufficient to produce durable therapeutic benefit. Combination strategies may therefore be worth exploring when supported by a clear mechanistic rationale. For example, pairing condensate-directed interventions with treatments that target complementary inflammatory, metabolic, or fibrogenic pathways. However, such combinations should be guided by cell-type- and disease-stage-specific evidence rather than applied broadly. Ultimately, candidate therapies will need to be evaluated in pharmacologically relevant models that assess efficacy, pharmacokinetics, tissue distribution, target engagement, and safety in parallel.

Therefore, a deeper understanding of the mechanism of action, regulators, and effectors that modulate LLPS is essential for rational anti-fibrosis drug development. The therapeutic goal is unlikely to be broad inhibition of phase separation, but rather selective modulation of disease-relevant condensates. These mechanistic insights into LLPS will inform several complementary strategies for therapeutic development: (i) design inhibitors or modulators that target specific amino-acid motifs, multivalent interaction interfaces, or post-translational modifications that govern phase separation; (ii) develop or engineer peptides that selectively promote or disrupt the LLPS behavior of disease-relevant proteins; (iii) use high-throughput screening and quantitative fluorescence imaging to identify small molecules that modulate condensate formation, dynamics, or material properties; and (iv) advance promising candidates through interdisciplinary evaluation of target engagement, efficacy, pharmacokinetics, tissue distribution, and safety. AI-assisted computational approaches may further facilitate compound screening, target identification, and optimization of candidate modulators.

Together, these considerations suggest that the next phase of the field should move from cataloging fibrosis-associated condensates toward establishing causal mechanisms and therapeutically actionable vulnerabilities. Defining when, where, and how specific MLOs or biomolecular condensates contribute to fibrosis will be essential for determining whether phase-separation biology can provide advantages beyond conventional pathway inhibition. We hope that this review helps clarify current knowledge gaps, highlights priorities for future mechanistic studies, and contributes to the exploration of more selective therapeutic approaches for organ fibrosis.

## Figures and Tables

**Table 1:** Organ Fibrosis-Related Membraneless Organelles.

MLOs/Condensates	Location	Scaffold/Core Protein	Client Component	Cell Types	Related Disease/Stress	Mechanism	Ref.
FOXK1 condensates	nucleus	FOXK1	RNA Pol II	Renal tubular epithelial cells (TECs): two types of proximal tubular epithelial cell lines including mouse BUMPT cells and human HK-2 cells	Chronic kidney disease (CKD)/TGF-β stimulation	FOXK1 IDRs interact with RNA Pol II forms to condense through LLPS, driving the transcription of glycolytic genes.	[46]
NONO condensates	nucleus	EWSR1	VGLL3	Myofibroblasts (cardiac myofibroblasts, HSCs and neonatal rat cardiac fibroblasts)	Cardiac fibrosis/TGF-β stimulation	VGLL3 undergoes LLPS via its low complexity domain and is incorporated into non-paraspeckle NONO condensates containing EWSR1. VGLL3 binds EWSR1 and suppresses miR-29b, which targets collagen mRNA.	[50]
CEBPZ condensates	nucleus	CEBPZ	Baicalein	Human hepatic stellate cells (HSCs): LX-2	Liver fibrosis/TGF-β stimulation	Baicalein directly binds CEBPZ and suppresses its LLPS. This enhances TP53 transcription and activates the p53/HK2 axis, leading to glycolytic inhibition, hepatic stellate cell (HSC) senescence, and attenuation of liver fibrosis.	[47]
hnRNP L condensates	nucleus	hnRNP L	\	Human fetal lung fibroblasts, Medical Research Council cell strain-5 (MRC-5)	Pulmonary fibrosis/TGF-β stimulation	Transforming growth factor-β (TGF-β) enhances hnRNP L phase separation, promoting profibrotic transcription and fibroblast-to-myofibroblast differentiation in pulmonary fibrosis.	[51]
PML/BRD4 condensates	nucleus	PML	BRD4	Human liver sinusoidal endothelial cells	Liver fibrosis/TNF-α treatment	PML and BRD4 form super-enhancer-associated condensates that activate proinflammatory angiocrine signaling and promote liver fibrosis.	[52]
Stress granule (SG)	cytoplasm	G3BP1/2	TIA1, FUS, PABP, DDX3X etc.	HL-1 atrial cells, cardiomyocytes, cardiac fibroblasts from SD rats	Atrial fibrillation/angiotensin II stimulation	G3BP1 overexpression inhibited cardiac fibroblast proliferation and reduced the protein expression levels of collagen I and fibronectin-1 in angiotensin II-stimulated cardiac fibroblasts.	[53]
YTHDF2 condensates	cytoplasm	YTHDF2	m^6^A modification of neurogenic locus notch homolog protein 1 (*NOTCH1*) mRNA	Mouse primary cardiac fibroblasts, H9C2 (rat cardiomyocytes) and 3T3 (mouse embryonic fibroblasts)	Diabetic cardiac fibrosis/high glucose combined with palmitic acid and oleic acid stimulation	m^6^A-dependent YTHDF2 phase separation enhances YTHDF2 recognition of *NOTCH1* mRNA and promotes NOTCH1 downregulation. Reduced NOTCH1 signaling promotes mitochondrial fission and aggravates diabetic cardiac fibrosis.	[35]
CBX7 condensates	cytoplasm	CBX7	ATP7A	Primary cardiomyocytes were isolated from young adult (3–6 months) and aged (24 months) C57BL/6 mice	Cardiac repair after myocardial ischemia–reperfusion/ischemia (0.5% O_2_, 5% CO_2_) for 1 h and reperfusion (21% O_2_) for 24 h	In aged myocardial ischemia–reperfusion hearts, CBX7 forms LLPS condensates with ATP7A, trapping ATP7A intracellularly and reducing copper efflux. δ-Amyrenone (δAe) disrupts CBX7–ATP7A condensation, restores ATP7A trafficking, reduces cuproptosis, and improves cardiac repair with less fibrosis and arrhythmia.	[54]
FSP1 condensates	cytoplasm	FSP1	\	AML12 cells, the human hepatocellular carcinoma Huh7 cells; primary mouse hepatocytes	Metabolic dysfunction-associated steatohepatitis (MASH)/PA to induce lipotoxicity	GSK3β inhibition or hepatocyte-specific GSK3β deletion reduces FSP1 phase separation in lipotoxic hepatocytes. This is associated with improved mitochondrial function, reduced ferroptosis, and attenuation of inflammation and fibrosis in MASH models.	[55]
Vimentin condensates	cytoplasm	Vimentin	\	Fibroblast-like African green monkey kidney cells; SW13/cL.2 cells; Human lung adenocarcinoma A549 cells	Oxidative stress-related diseases/H_2_O_2_ treatment	The transition of vimentin filaments into condensates under oxidative stress requires C328. Vimentin droplets contain low-complexity domain interactions that are altered by 1,6-hexanediol and may act as reserves for filament recovery after oxidative stress.	[56]
RXRα condensates	cytoplasm	RXRα	CaMKKβ and AMPKα	Human primary hepatic stellate cells; mouse primary hepatic stellate cells; LX-2 cells	Liver fibrosis/TGF-β stimulation	K-80003 activates AMPKα by inducing RXRα to form condensates with CaMKKβ and AMPKα via a two-phase process. The formation of RXRα condensates is driven by its N-terminal intrinsic disorder region and requires phosphorylation by CaMKKβ.	[33]
LIGs	cytoplasm	DDX49	m^5^C-modified Timp2 mRNA	Human hepatocyte cell line HHL5; Mouse primary hepatocytes; non-parenchymal cells (NPCs) and LX-2 cells	Metabolic dysfunction-associated steatotic liver disease (MASLD)/NaAsO_2_, H_2_O_2_, OA/PA treatment; MCD medium	Arachidonic acid metabolites induce the formation of LIGs assembled by the scaffold DDX49 to sequester m^5^C-modified Timp2 mRNA, thereby suppressing the production of the profibrotic hepatokine TIMP2 and alleviating fibrosis progression in steatotic liver.	[34]
PrPC condensates	cytoplasm	PrP^C^	TBK1	PTECs and fibroblasts	CKD/TGF-β stimulation	PrPC undergoes LLPS in proximal tubular epithelial cells and activates the TBK1–IRF3 axis. This condensate-linked inflammatory signaling promotes profibrotic responses in tubular cells and fibroblasts, whereas PrPC loss or TBK1 inhibition attenuates renal fibrosis.	[48]
Ferritin condensates	cytoplasm	ferritin	NCOA4	Human lung fibroblast MRC-5 cell line and the murine alveolar epithelial cell line MLE12	Pulmonary fibrosis/mechanical stretch; DFO treatment	Mechanical stress upregulates NCOA4 in alveolar epithelial cells and enhances ferritin phase separation. NCOA4-driven ferritin condensation promotes ferritinophagy, ferroptosis, and ferritin-containing EV release, thereby contributing to mechanical ventilation-induced pulmonary fibrosis (MVPF).	[57]
PCM1 condensates	cytoplasm/centrosome	PCM1	CEP131	Non-tumorigenic human mammary epithelial cell line MCF10A cells	Pulmonary fibrosis/OSMI-1or TMG treatment	O-GlcNAcylation of PCM1 and CEP131 promotes their phase-separation-dependent localization to centrosomes. Loss of O-GlcNAcylation disperses these proteins, disrupts centrosome–microtubule coupling and epithelial polarity, and exacerbates pulmonary fibrosis.	[58]
TRIM25 condensates	cytoplasm	TRIM25	circELP2 14-3-3ζ	Human lung fibroblast, MRC-5 cell line	Pulmonary fibrosis/TGF-β stimulation	circELP2 promotes TRIM25 phase separation, leading to YAP1 nuclear translocation, cytoskeletal remodeling, and fibroblast-to-myofibroblast differentiation.	[59]
Ras-related protein Rab-18 (RAB18) condensates	cytoplasm/mitochondrial-associated membrane	RAB18	\	HSCs (primary mouse HSCs, LX2)	Liver fibrosis/DATs treatment	Diallyl trisulfide (DAT) induces RAB18 phase separation and promotes mitochondrial-associated membrane formation in HSCs. RAB18 condensation inhibits lipophagy and triggers cuproptosis, producing antifibrotic effects in liver fibrosis models.	[60]
SORBS3 condensates	cytoplasm/plasma membrane	SORBS3	FBXO2	Primary mouse hepatocyte; C2C12 cell line; LX-2; mouse hepatoma cell line Hepa1-6	Liver fibrosis/lactate treatment	SORBS3 lactylation promotes SORBS3 LLPS and strengthens its interaction with flotillin-1, enabling selective FBXO2 loading into small extracellular vesicles termed lactate bodies. Lactate bodies induce hepatocyte apoptosis and subsequent HSC activation through the MCL1–BAX/BAK axis, promoting overtraining-associated liver fibrosis.	[61]
WNK1-TPI1 condensates	cytoplasm	WNK1	TPI1	Primary human HSCs	Liver fibrosis/PDGF treatment	WNK1 interacts with TPI1 in condensates to enhance TPI1-mediated glycolytic signaling and HSC activation in liver fibrosis.	[62]
DDR1 condensates	plasma membrane	DDR1	LATS1	Human umbilical artery smooth muscle cells; NIH3T3 cells	Arterial stiffness/serum-free and soluble collagen I treatment	Matrix stiffness or collagen binding promotes DDR1 phase separation and co-condensation with LATS1. DDR1–LATS1 condensates disrupt interaction of LATS1 and MOB1, inactivate Hippo signaling, activate YAP, and drive arterial ECM remodeling and stiffening.	[49]
FGFBP1 condensates	plasma membrane	FGFBP1	FGFs heparan sulfate	Primary mouse myoblasts	Acute liver injury (ALI)/Dexamethasone administration	FGF6 regulates the heparin-dependent release kinetics of FGFBP1 by perturbing its LLPS-driven condensate dynamics at the plasma membrane during liver regeneration.	[63]

**Table 2:** Therapeutic Targeting and MLOs.

Small Molecules/Peptides	MLOs/Condensates	Related Diseases	In Vivo Results	Mechanism	Ref.
Baicalein	CEBPZ condensates	Liver fibrosis	In CCl_4_-induced hepatic fibrotic mice, baicalein alleviated liver fibrosis, reduced inflammatory response and collagen deposition, triggered hepatic stellate cell senescence, and suppressed glycolysis.	Baicalein suppresses nuclear CEBPZ phase separation, enhances *TP53* transcription, inhibits the p53/HK2 glycolytic program, and promotes hepatic stellate cell senescence, thereby attenuating liver fibrosis.	[47]
K-80003	RXRα condensates	Liver fibrosis	In CCl_4_-injured and AMLN-diet-fed fibrotic mice, pharmacological activation of RXRα suppressed hepatic stellate cell activation and mitigated liver fibrosis.	K-80003 promotes formation of RXRα-CaMKKβ-AMPKα condensates, enhancing AMPKα activation and mitophagy and suppressing HSC activation and liver fibrosis.	[33]
Diallyl trisulfide (DATS)	RAB18 condensates	Liver fibrosis	In fibrotic rats, garlic-derived diallyl trisulfides restrained liver fibrogenesis by triggering cuproptosis and repressing lipophagy of activated hepatic stellate cells.	The garlic-derived compound induces phase separation of RAB18, selectively protects hepatocytes while inhibiting lipophagy and triggering cuproptosis in hepatic stellate cells (HSCs).	[60]
iBET151	PML/BRD4 condensates	Liver fibrosis	In fibrotic mouse livers, endothelial-specific deletion of PML or BRD4 inhibition abrogated PML-BRD4 condensate-driven hepatic inflammation, macrophage infiltration, and subsequent liver fibrosis.	iBET151, a pharmacological BRD4 inhibitor, suppresses BRD4 phase separation and diminishes super enhancer activity, reducing hepatic inflammation and fibrosis.	[52]
WNK-IN-11	WNK1 condensates	Liver fibrosis	In CCl_4_- and choline-deficient L-amino-acid-diet-treated mice, genetic or pharmacological WNK1 inhibition in hepatic stellate cells blunted liver fibrosis.	WNK-IN-11 is a pharmacological inhibitor of WNK1 that attenuates liver fibrosis in mice and in the human PCLS model.	[62]
δ-Amyrenone (δAe)	CBX7 condensates	Aging diseases	In aged myocardial-infarction mice and Bama minipigs, CBX7-inhibitor-loaded multifunctional hydrogel ameliorated cardiac remodeling and restored cardiac function by regulating CBX7-dependent cuproptosis.	As a selective CBX7 inhibitor that disrupts CBX7-ATP7A LLPS, restores ATP7A trafficking and copper efflux, and improves cardiac function while reducing fibrosis and arrhythmias.	[54]
Amlexanox	PrPC aggregation	CKD	In UUO, UIRI, and chronic kidney disease mouse models, genetic ablation of cellular prion protein or TBK1 pharmacological blockade mitigated renal interstitial fibrosis and protected renal function.	Amlexanox reduces the PrPC ability of phase separation by increasing phosphorylation of TBK1 on Ser172 and thus blocking phosphorylation of IRF3, attenuating renal fibrosis in mouse CKD models.	[48]
MCC950 inhibitor	NLRP3 condensates	Inflammation-related diseases	In LPS-challenged septic mice, ZDHHC7 deficiency or palmitoylation inhibitor treatment diminished NLRP3-phase-separation-dependent IL-1β secretion and improved mouse survival upon LPS stimulation.	The signal-induced NLRP3 phase separation likely provides the simplest and most direct mechanistic basis for NLRP3 inflammasome activation. MCC950 could prevent NLRP3 conformational changes and phase separation.	[80]
Aminosterol claramine	Aggregated forms of α-synuclein constitute the major component of Lewy bodies.	Parkinson’s disease	In α-synuclein-overexpressing *Caenorhabditis elegans*, the aminosterol claramine suppressed the maturation of α-synuclein amyloid aggregates within liquid condensates and rescued worm motor deficits.	Aminosterol claramine stabilizes α-synuclein condensates and inhibits primary nucleation within the condensates both in vitro and in a *Caenorhabditis elegans* model of Parkinson’s disease.	[81]
G3BP inhibitor a and b (G3Ia and G3Ib)	Stress granule	Neurodegenerative disease	N/A	These compounds inhibit stress granule formation in cells treated before or concurrent with stress and dissolve pre-existing stress granules. These effects are consistent across multiple cell types and a variety of initiating stressors.	[82]
A cell-penetrating peptide	CNBP condensates	Neuroblastoma (NB)	In neuroblastoma-bearing mice, peptide-mediated disruption of CNBP phase separation impeded tumor growth, restrained ribosome biogenesis, and reprogrammed the tumor immune microenvironment.	CNBP condensates repressed the activity of switch/sucrose-nonfermentable (SWI/SNF) core subunits (SMARCC2/SMARCC1/SMARCA4) via interaction with SMARCC2, leading to an increased activity of the SMARCC1/SMARCA4 binary complex, which facilitates gene expression essential for 18S ribosomal RNA (rRNA) processing in tumor cells. A cell-penetrating peptide blocking phase separation and interaction of CNBP with SMARCC2 inhibited ribosome biogenesis and NB progression.	[83]
Peptides designed specifically for non-erythrocytic αII-spectrin (SPTAN1)	SPTAN1 condensates	HBV-related HCC	In hepatocellular carcinoma xenograft mice, inhibitory peptide against SPTAN1 lactylation retarded tumor progression, dampened NOTCH1/HES1 signaling, and reversed intratumoral immunosuppression.	Lactylated SPTAN1 as a novel oncogenic driver in HBV-related HCC, functioning via metabolic reprogramming and immune modulation.	[84]
HSP90 inhibitor 17-AAG	Albumosomes	Nonalcoholic fatty liver disease (NAFLD)	In high-fat-diet-fed and aged mice, enhanced formation of cytoplasmic albumosomes stabilized mitochondrial homeostasis and protected mice against the development of nonalcoholic fatty liver disease.	Endogenous pre-folding albumin undergoes phase separation in the cytoplasm to form albumosomes. Albumosomes maintain mitochondrial homeostasis from exhaustion by interacting with CPT2. Physiologically, albumosomes accumulate in hepatocytes during murine aging and protect the livers of aged mice from mitochondrial damage and fat deposition. The HSP90 inhibitor 17-AAG promotes hepatic albumosomal accumulation in vitro and in vivo, thereby suppressing the progression of NAFLD in mice.	[85]

N/A, not available.

## Data Availability

No new data were created or analyzed in this study. Data sharing does not apply to this article.
